# Ancient Endogenous Pararetroviruses in *Oryza* Genomes Provide Insights into the Heterogeneity of Viral Gene Macroevolution

**DOI:** 10.1093/gbe/evy207

**Published:** 2018-09-18

**Authors:** Sunlu Chen, Nozomi Saito, Jaymee R Encabo, Kanae Yamada, Il-Ryong Choi, Yuji Kishima

**Affiliations:** 1 Laboratory of Plant Breeding, Research Faculty of Agriculture, Hokkaido University, Sapporo, Japan; 2 State Key Laboratory of Crop Genetics and Germplasm Enhancement, College of Agriculture, Nanjing Agricultural University, Nanjing, China; 3 Rice Breeding Platform, International Rice Research Institute, Los Baños, Laguna, Philippines; 4 Microbiology Division, Institute of Biological Sciences, University of the Philippines Los Baños, Los Baños, Laguna, Philippines

**Keywords:** genomic fossil, paleovirology, endogenous pararetrovirus, viral gene macroevolution, rate heterogeneity, *Oryza*

## Abstract

Endogenous viral sequences in eukaryotic genomes, such as those derived from plant pararetroviruses (PRVs), can serve as genomic fossils to study viral macroevolution. Many aspects of viral evolutionary rates are heterogeneous, including substitution rate differences between genes. However, the evolutionary dynamics of this viral gene rate heterogeneity (GRH) have been rarely examined. Characterizing such GRH may help to elucidate viral adaptive evolution. In this study, based on robust phylogenetic analysis, we determined an ancient endogenous PRV group in *Oryza* genomes in the range of being 2.41–15.00 Myr old. We subsequently used this ancient endogenous PRV group and three younger groups to estimate the GRH of PRVs. Long-term substitution rates for the most conserved gene and a divergent gene were 2.69 × 10^−8^ to 8.07 × 10^−8^ and 4.72 × 10^−8^ to 1.42 × 10^−7^ substitutions/site/year, respectively. On the basis of a direct comparison, a long-term GRH of 1.83-fold was identified between these two genes, which is unexpectedly low and lower than the short-term GRH (>3.40-fold) of PRVs calculated using published data. The lower long-term GRH of PRVs was due to the slightly faster rate decay of divergent genes than of conserved genes during evolution. To the best of our knowledge, we quantified for the first time the long-term GRH of viral genes using paleovirological analyses, and proposed that the GRH of PRVs might be heterogeneous on time scales (time-dependent GRH). Our findings provide special insights into viral gene macroevolution and should encourage a more detailed examination of the viral GRH.

## Introduction

Endogenous viral elements (EVEs) in eukaryotic genomes have arisen from the vertical inheritance of viral sequences that have occasionally been integrated into host germline genomes (Feschotte and Gilbert 2012; Aiewsakun and Katzourakis 2015a). Although natural virus fossils are unavailable, EVEs can serve as genomic fossils of ancient exogenous viruses (Feschotte and Gilbert 2012; Katzourakis 2013; Chen and Kishima 2016). The mining and analysis of EVEs has had an important impact on the emerging field of paleovirology, with implications for the characterization of viral origins and evolution as well as host–virus and virus–virus coevolution (Patel et al. 2011; Feschotte and Gilbert 2012; Aiewsakun and Katzourakis 2015a; Chen et al. 2017).

Viral evolutionary rates are heterogeneous in many respects, including across virus groups and time scales (Duffy et al. 2008; Gibbs et al. 2010; Duchêne et al. 2014; Aiewsakun and Katzourakis 2016). Most viral genomes comprise multiple genes exhibiting different degrees of sequence conservation or evolutionary divergence, which indicates an evolutionary rate heterogeneity between different viral genes (gene rate heterogeneity, GRH). This viral GRH is a consequence of the diverse evolutionary forces and selection pressures imposed on different viral genes. In particular, some core viral genes, such as those encoding reverse transcriptases (RTs) (Xiong and Eickbush 1990), are highly conserved. Such conserved genes with core functions related to viral replication evolve under high functional constraints (Miyata et al. 1980; Fay and Wu 2003; Knipe and Howley 2013). Many viral genomes encode one or more additional genes that are often highly divergent and have no obvious homology to other known genes (species/genus-specific). These divergent genes reportedly increase viral adaptation and fitness to hosts or vectors and evolve with high adaptive plasticity (Miyata et al. 1980; Fay and Wu 2003; Knipe and Howley 2013). Although conserved genes obviously evolve more slowly than divergent genes, GRH in a viral genome has not been examined on a long-term scale and thus cannot presently be compared with short-term GRH. EVEs have recently been applied for estimating viral long-term substitution rates (Gilbert and Feschotte 2010; Lefeuvre et al. 2011; Suh et al. 2013), which, together with many other findings, has drawn attention to the time-dependent rate phenomenon of viruses (viral rate heterogeneity on time scales) (Gibbs et al. 2010; Wertheim and Kosakovsky Pond 2011; Duchêne et al. 2014; Aiewsakun and Katzourakis 2015b, 2016; Lin et al. 2015). Suitable EVEs can also be used to estimate the unknown long-term GRH of exogenous viruses, which may help to clarify viral adaptive evolution.

Plant pararetroviruses (PRVs) comprising the family *Caulimoviridae* are reverse-transcribing, double-stranded DNA viruses (Temin 1985) that represent a serious threat to global plant health and production. For example, the rice tungro bacilliform virus (RTBV) infecting rice (*Oryza sativa*) is mainly responsible for rice tungro disease, which has considerably affected rice production in South and Southeast Asia (Hull 1996; Azzam and Chancellor 2002). All known extant PRVs contain four conserved genes that encode a movement protein (MP), a capsid protein (CP), a protease (PR), and an RT with RNase H activity (RT/RH), all within a single long open reading frame (ORF) (e.g., RTBV) or multiple short ORFs (e.g., cauliflower mosaic virus, CaMV) (Hohn and Rothnie 2013). PRVs often also include various relatively divergent genes that may influence multiple processes, including vector transmission and immune suppression (Hohn 2013; Hull 2014). Although lacking an integrase and a process for integration, PRVs possess EVEs known as endogenous PRVs that originated from nonhomologous end-joining between PRVs and host genomes (Liu et al. 2012). As the most abundant known EVEs in plant genomes (Hohn et al. 2008; Geering et al. 2014; Diop et al. 2018; Gong and Han 2018), endogenous PRVs may enable the study of long-term GRH in viruses.

Molecular dating of EVEs (i.e., determination of the endogenization time of an EVE in host genomes) underlies many aspects of EVE-assisted viral macroevolution studies. Robust dating of endogenous PRVs can be achieved by analyzing orthologs using a series of related host species with a known phylogeny (Feschotte and Gilbert 2012; Chen and Kishima 2016). The endogenous RTBV-like (eRTBVL) family, which is an endogenous PRV family similar to RTBV, has been identified in rice genomes (Kunii et al. 2004; Chen et al. 2014). As endogenous PRVs that are not reactivated after endogenization, eRTBVLs are a good model system for investigating PRV paleovirology (Chen and Kishima 2016). The most ancient eRTBVL group is eRTBVL-D, which was endogenized long before the speciation of rice (Chen et al. 2014). In this study, we obtained precise molecular dates for eRTBVL-D segments and used them to estimate long-term nucleotide substitution rates for PRV genes. Rate comparisons revealed that the long-term GRH between the most conserved PRV gene and a divergent gene corresponded to an almost 1.83-fold difference in substitution rates, which is lower than that observed for short-term GRH according to published data. Different viral genes exhibit heterogeneity regarding evolutionary rates. We identified in this study focusing on long-term evolutionary scales an additional time dependent-factor influencing diversity of viral GRH.

## Materials and Methods

### Genomic Screening of eRTBVL-D Segments

The systematic screening of eRTBVL-D segments involved an initial BLASTn search of the *O. sativa japonica* genome with the BLAST+ 2.2.27 tool (Camacho 2009), with six previously identified segments as queries (Liu et al. 2012; Chen et al. 2014). Segments from other eRTBVL groups were removed from the highly reliable hits (*e*-values <1 × 10^−3^ and lengths >100 bp). We then conducted a BLASTn search of the rice genome using the consensus sequences of other eRTBVL groups as queries (Chen et al. 2014). Because the sequences of these young, non-eRTBVL-D groups were highly similar, we only extracted highly reliable hits (*e*-values < 1 × 10^−3^ and lengths >100 bp) exhibiting < 85% sequence identities to non-eRTBVL-D consensus sequences. The segments from the two rounds of screening were collectively designated as the eRTBVL-D group (all identified segments have *e*-values < 1 × 10^−8^ during screenings) and were mapped to the reconstructed viral genomes. We also performed a tBLASTn search using the translated protein sequences of the eRTBVL groups, and additional eRTBVL-D segments >100 bp were not detected. In case some genomic loci contained multiple eRTBVL-D segments (one segment next to or near another segment), potentially with independent origins or rearrangement events, individual segments were detected based on analyses of viral genomic structure and synteny of integration sites (see the following Genome-Wide Orthology Analysis section). All detected segments underwent these analyses.

### Genome-Wide Orthology Analysis

Available genome assembly data of *Oryza* species were acquired primarily from the Gramene database (Tello-Ruiz et al. 2016). Details of assembly sources and versions used in the study are provided in supplementary table S1, Supplementary Material online. The left and right 5-kb flanking sequences of each identified eRTBVL-D locus in the *O. sativa japonica* genome were mapped onto other *Oryza* genomes using BLASTn. Because *O. sativa* and *O. glaberrima* were domesticated from *O. rufipogon* and *O. barthii*, respectively (Huang et al. 2012; Wang et al. 2014), we also conducted an orthology analysis using the genomes of those ancestral species. This analysis yielded results identical to those obtained from the *O. sativa* and *O. glaberrima* genomes (supplementary table S2, Supplementary Material online). The mapping results were rechecked using available genome collinearity data (genome-wide alignments between *Oryza* genomes) from the Gramene database (Tello-Ruiz et al. 2016).

### Plant Materials

Seeds of the studied *Oryza* species were obtained from the National Institute of Genetics (Japan) and the International Rice Research Institute. Accession numbers are listed in supplementary table S3, Supplementary Material online. *O. rufipogon* and *O. barthii* accessions were also examined. Sterilized seeds of all accessions were germinated in culture dishes, and 1-week-old seedlings were transferred to pots containing soil, and then incubated in a greenhouse at Hokkaido University. Leaf samples were collected for total DNA extraction using cetyltrimethylammonium bromide DNA extraction buffer. The extracted DNA samples were quantified using a NanoDrop 2000 instrument (Thermo Fisher Scientific), and the concentrations were adjusted to similar levels.

### PCR Amplification and Sequencing

For the PCR amplification and sequencing of *Oryza* DNA, the following three sets of primers were generally designed for each eRTBVL-D segment: for amplification A, a left-flanking forward primer and a reverse primer within the eRTBVL-D segment; for amplification B, a forward primer within the eRTBVL-D segment and a right-flanking reverse primer; and for amplification C, left- and right-flanking primers. The PCR amplifications were completed using Ex *Taq* or LA *Taq* polymerase (Takara) and a PTC-200 thermal cycling system (GMI). The PCR program was as follows: 94°C for 3 min; 32 cycles of 94°C for 30 s, 48–58°C for 30 s, and 72°C for 0.5–3 min; 72°C for 5–10 min. The PCR products were separated by 1–2.5% agarose gel electrophoresis. The gels were stained with GelRed (Biotium) and visualized under UV light with an AE-6933FXES Printgraph system (ATTO). The examined PCR products were purified for sequencing with a NucleoSpin Gel or PCR Clean-up kit (Takara). Sanger sequencing was completed using a BigDye Terminator v3.1 cycle sequencing kit (Applied Biosystems) and an ABI 3730 DNA Analyzer (Applied Biosystems). The Seqscanner version 1.0 program (Applied Biosystems) was used for sequence calling and quality assessment. Final sequences were assembled using the Jemboss toolkit (Carver and Bleasby 2003). Details regarding the primers used in this study are provided in supplementary table S4, Supplementary Material online.

### Tests of Neutral Evolution

For neutrality tests of eRTBVL-D orthologous sequences, we first completed the d*N*/d*S* ratio test (nonsynonymous substitution rate vs synonymous substitution rate) for the coding regions (domain/ORF). Alignments for each orthologous data set were generated in MUSCLE (Edgar 2004) and then edited manually. Maximum likelihood estimates of substitution rates and statistical significance tests for each site (codon) in each data set were conducted with the HyPhy program (Pond et al. 2005). The HKY model was chosen for all data sets after the substitution model test. We subsequently conducted codon-based Fisher’s exact test of selection for each pair of orthologous sequences in each data set (Zhang et al. 1997). The numbers of synonymous and nonsynonymous differences between orthologous sequences were estimated by the modified Nei–Gojobori method (Zhang et al. 1998). All analyses were completed with MEGA version 7.0 (Kumar et al. 2016). Additionally, we employed two methods based on the site frequency spectrum (Tajima’s *D* [Tajima 1989] and Fu and Li’s D*/F* [Fu and Li 1993]) to further analyze the respective whole-length and domain/ORF alignments. These tests and statistical analyses were completed using DnaSP version 5.10 (Librado and Rozas 2009) with default parameters. The possibility of domestication-derived effects was excluded by performing analyses as described earlier using the progenitor species.

### Genetic Distance and Substitution Rate Analyses

We estimated genetic distances between the viral sequences of the oldest eRTBVL-D group and three younger eRTBVL groups (eRTBVL-A, -B, and -C; 0.01–0.16 Myr old). For the younger eRTBVL groups, the corresponding viral genomic sequences were represented by consensus sequences constructed from multiple copies of each group (Chen et al. 2014). However, no consensus sequence could be constructed for the eRTBVL-D group because of an insufficient number of high-quality copies. To simplify and enable the calculation, we ignored the time required for a viral sequence inserted into a host germline genome to be endogenized in a host population. This time period is actually unknown, but is essentially zero relative to a million years of macroevolution (Gilbert and Feschotte 2010). Given this assumption, the distance between eRTBVL-D sequences and the viral sequences of eRTBVL-A/-B/-C (consensus sequences) could be divided into the following two components: variations between viral sequences of eRTBVL-D and eRTBVL-A/-B/-C, and mutations of eRTBVL-D sequences accumulated in the host genome since endogenization. Consequently, we first calculated pairwise distances (uncorrected distances) between eRTBVL-D sequences in the *O. sativa japonica* genome and eRTBVL-A/-B/-C consensus sequences. The pairwise distances were then corrected by subtracting the distance corresponding to the number of accumulated mutations in host genomes since eRTBVL-D endogenization (2.41–6.76 Myr for the examined segments). The accumulated distances of eRTBVL-D segments in rice genomes were estimated as half the distance between orthologous *O. sativa japonica*/*O. meridionalis* eRTBVL-D sequences (these accumulated distances are 0.007, 0.006, 0.005, 0.006 for the d3, d10, d11, and d14 segments, respectively).

Pairwise distances were calculated with MEGA version 7.0 (Kumar et al. 2016). After generating alignments in MUSCLE (Edgar 2004) followed by manual editing, the best nucleotide substitution models for each data set were determined with MEGA based on the Bayesian information criterion. TN93+G was selected as the best model for all whole-length data sets. The best models for the whole-length data sets were also selected for the data sets of the respective domains/ORFs. To verify that the previously constructed consensus sequences were good representatives of their corresponding viral genomic sequences (Chen et al. 2014), we also calculated the distances between eRTBVL-D sequences and the raw sequences of eRTBVL-A/-B/-C (supplementary table S5, Supplementary Material online). The resulting corrected distance divided by the divergence time between the viral sequences of the studied eRTBVL-D and eRTBVL-A/-B/-C segments (approximated as the difference between the ages of eRTBVL-D and eRTBVL-A/-B/-C sequences [2.25–6.75 Myr] when the time required for endogenization of a viral sequence inserted into a host germline genome was ignored) was equal to the substitution rate during the designated period. The GRH between two viral genes was quantified as the fold difference between their substitution rates.

## Results and Discussion

### Segments of the eRTBVL-D Group in the Rice Genome

**Fig. 1.**
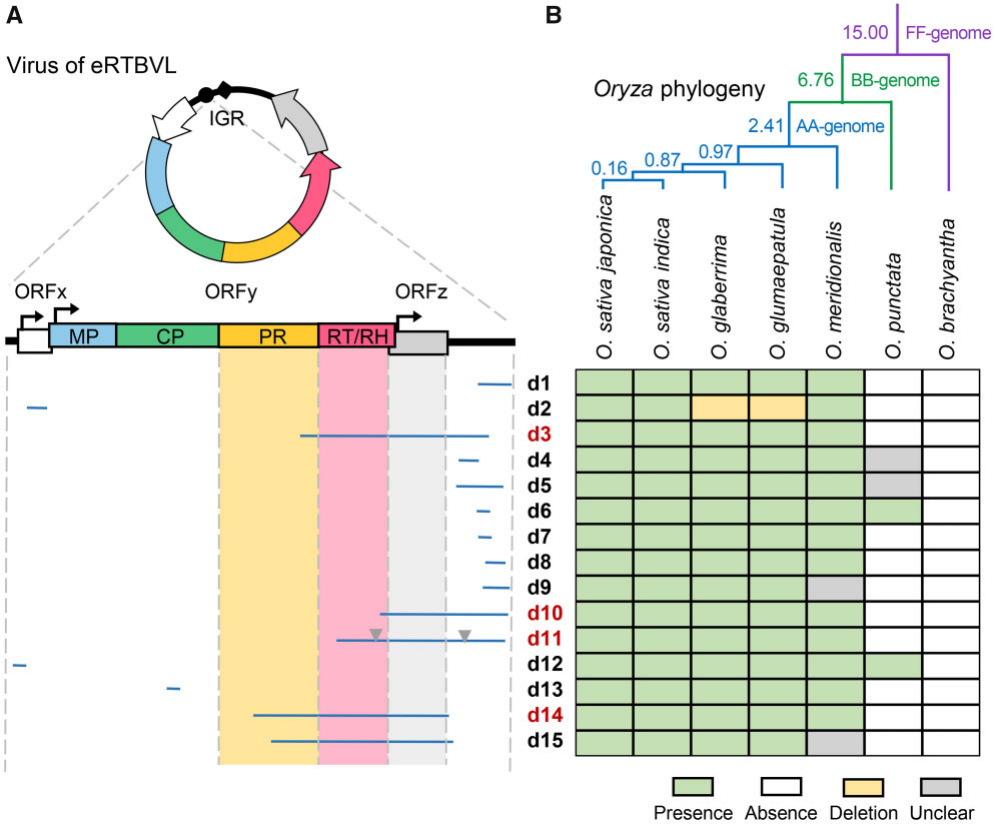
—Viral genomic structure of eRTBVL-D segments in the *Oryza sativa* genome (*A*) and ortholog presence/absence in the genus *Oryza* (*B*). In panel (*A*), the circular viral genome is displayed at the top with open reading frames (ORFs) represented with arrows and functional domains (genes) outlined in different colors. Intergenic regions (IGRs) are represented as black curved lines. Black dots and diamonds indicate primer-binding sites and polypurine tracts, respectively. The eRTBVL-D segments were mapped to the linear viral genome, where ORFs are indicated by rectangles with arrows, and IGRs are represented by thick black lines. Domains/ORFs (genes) examined in detail in this study are highlighted, and segments examined in detail are indicated by red IDs. Two large insertions in the d11 segment are indicated by inverted triangles. In panel (*B*), the known phylogeny of the genus *Oryza* (Zheng and Ge 2010; Huang et al. 2012; Stein et al. 2018) is presented at the top. Branches corresponding to *Oryza* AA-, BB-, and FF-genome groups are depicted in different colors, and their corresponding divergence times (millions of years) are labeled. The table summarizes the pattern of ortholog presence/absence of each eRTBVL-D segment. Green, white, and gray indicate presence, absence, and unclear results, respectively, with yellow symbolizing loss due to deletion. Detailed results are provided in supplementary table S2, Supplementary Material online.

Systematic screening of the rice genome using known sequences revealed 15 eRTBVL-D segments >100 bp (table 1), six of which were detected in a previous study (Chen et al. 2014). Twelve of these segments were mapped to the second half of the linear viral genome, mainly in intergenic regions (IGRs) (fig. 1*A*). It is unclear why the majority of these segments were derived from IGRs or why there were more segments in the second half of the linear genome than in the first half. The pregenomic RNA and open-circular genomes of PRVs are terminally redundant at IGRs (Qu et al. 1991; Hull 1996; Hohn and Rothnie 2013), which may be related to the relatively high frequency of IGR-derived segments.

**Table 1:** Summary of eRTBVL-D Segments in the *Oryza sativa* Genome

ID	**Previous Name**^a^	Positions in Rice Genome				**Positions in Viral Genome**^b^		Length (bp)	**Disruptive Mutation Number**^c^	Distances to Neighbor Genes	
		Chr	Start	End	Strand	Start	End			Left	Right
d1	JaE1-4	1	5,193,920	5,194,407	+	6,880	7,353	488	NA	43,579	7,455
d2	JaE1-4	1	5,194,744	5,195,008	+	108	359	265	0	44,403	6,854
d3	JaE1-4	1	5,195,124	5,198,055	+	4,259	7,180	2,932	9	44,783	3,807
d4	NA	2	12,120,385	12,120,601	+	6,540	6,758	217	NA	5,419	7,312
d5	NA	2	12,120,644	12,121,245	−	6,539	7,184	602	NA	5,678	6,668
d6	NA	4	18,488,307	18,488,450	+	6,873	7,021	144	NA	8,092	1,903
d7	NA	4	18,488,544	18,488,674	−	6,852	6,984	131	NA	8,329	1,679
d8	NA	4	21,097,520	21,097,778	−	6,952	7,196	259	NA	86,783	85,199
d9	NA	5	6,506,720	6,507,050	−	6,953	7,278	331	NA	8,332	7,696
d10	JaE7-4	7	8,906,209	8,907,980	+	5,547	7,343	1,772	5	27,304	18,154
d11	JaE7-5/7-6	7	8,909,007	8,920,257	−	4,817	7,341	11,251	9	30,102	5,877
d12	NA	9	16,726,920	16,727,025	+	32	137	106	0	5,997	1,153
d13	NA	10	9,099,571	9,099,688	−	2,584	2,696	118	1	5,282	2,080
d14	JaE11-2	11	5,069,912	5,072,964	+	3,580	6,416	3,053	19	5,378	243
d15	JaE11-5	11	11,654,495	11,657,114	−	3,806	6,413	2,620	15	2,608	4,216

a Previous names of six segments (JaE1-4, 7-4, 7-5, 7-6, 11-2, and 11-5) are from Liu et al. (2012).

b Segments were mapped to the reconstructed viral genomes.

c Single nucleotide polymorphisms and insertion/deletion-induced nonsense mutations and frameshift mutations.

NA, not available for intergenic regions; Chr, chromosome.

### Ancient Endogenization of eRTBVL-D Segments in the Genus *Oryza*

For exact molecular dating of eRTBVL-D segments, we examined the presence/absence pattern of eRTBVL-D loci in other *Oryza* species. We completed a genome-wide orthology analysis of eRTBVL-D segments using available genomic data (supplementary table S1, Supplementary Material online) as well as a PCR analysis followed by Sanger sequencing (supplementary table S3, Supplementary Material online). According to the combined results, two short loci (d6 and d12) were endogenized before the speciation of *O. punctata* (*Oryza* BB-genome group), but after that of *O. brachyantha* (*Oryza* FF-genome group), whereas most loci were endogenized in the latest common progenitor of the *Oryza* AA-genome group (i.e., before the speciation of *O. meridionalis*, but after that of *O. punctata*) (fig. 1*B*; details in supplementary table S2, Supplementary Material online). The divergence times of *Oryza* species are well documented, and a very recent and comprehensive estimation based on large data sets has established the divergence times of *O. meridionalis*, *O. punctata*, and *O. brachyantha* as 2.41, 6.76, and 15.00 Ma, respectively, similar to values obtained in previous studies (Stein et al. 2018). Thus, the oldest eRTBVL-D segments can be traced to 6.76–15.00 Ma, whereas most segments are 2.41–6.76 Myr old (fig. 1*B*). These results imply that rice PRVs originated earlier than previously estimated (Chen et al. 2014).

### Long-Term Substitution Rates of PRVs

We selected four eRTBVL-D segments (d3, d10, d11, and d14) with characteristics that made them good candidate genomic fossils for paleovirological studies (i.e., clearly defined ages, ample lengths [>1,772 bp], and coverage of different viral genic regions [domains/ORFs]) (fig. 1). These eRTBVL-D segments contained multiple disruptive mutations (table 1), suggesting the absence of selection by hosts. Different tests of neutral evolution conducted for each ortholog data set of the four eRTBVL-D loci supported the assumption that all four segments evolved neutrally in their host genomes (supplementary table S6, Supplementary Material online). Therefore, we used the sequences of the four eRTBVL-D loci to calculate PRV long-term substitution rates.

**Fig. 2.**
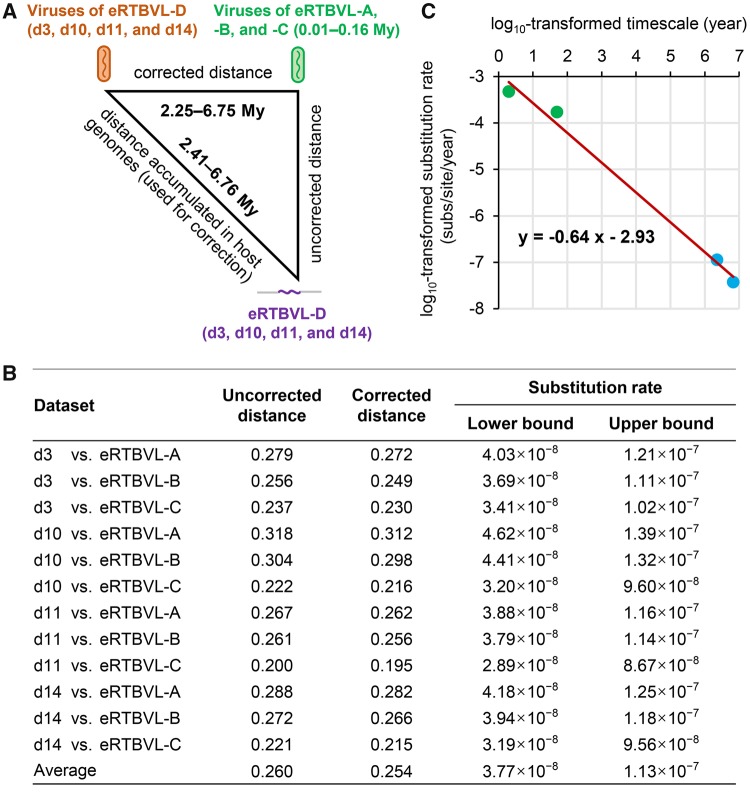
—Long-term substitution rates of plant pararetroviruses (PRVs) estimated with eRTBVL-D. (*A*) Strategy used to estimate genetic distances for substitution rate calculations. The distance between an eRTBVL-D sequence in the rice genome and viral sequences of eRTBVL-A/-B/-C (uncorrected distance) minus the distance accumulated in the rice genome from 2.41 to 6.76 Myr for eRTBVL-D was considered to approximate the distance between the viruses of eRTBVL-D and eRTBVL-A/-B/-C from 2.25 to 6.75 Myr (corrected distance). Each element is represented by a different color. The (unknown) amount of time required for a viral sequence to be endogenized in a host population was ignored because it was ∼0 relative to a million years of macroevolution. Therefore, the divergence time between the viral sequences of the studied eRTBVL-D and eRTBVL-A/-B/-C segments was approximated as the difference between the ages of eRTBVL-D and eRTBVL-A/-B/-C sequences. (*B*) Long-term substitution rates of PRVs calculated using corrected distances. (*C*) Time-dependent rate phenomenon of PRVs. The plot presents the relationship between substitution rates (substitutions/site/year) and the corresponding measurement time scales (years). The log_10_-transformed values underwent a linear regression analysis (red line), and the resulting equation is displayed. The data are from previous studies (short-term; green dots) (Yasaka et al. 2014; Guimarães et al. 2015) and from this study (long-term; blue dots). One value from Yasaka et al. (2014) that was calculated only from divergent gene regions was not included.

We previously revealed that the virus of eRTBVL was an ancient sister rather than an ancestor of extant RTBV, and that consensus sequences constructed from multiple copies of the relatively young eRTBVL groups (eRTBVL-A, -B, and -C endogenized in *O. sativa* ∼0.01–0.16 Ma) were very good representatives of the corresponding viral genomic sequences (Chen et al. 2014). A consensus sequence could not be constructed for the eRTBVL-D group because of a lack of high-quality copies. Consequently, we compared eRTBVL-D sequences with the reconstructed viral sequences of eRTBVL-A, -B, and -C for calculations (fig. 2*A*; for details, see Materials and Methods). In our calculations, we ignored the time required for a viral sequence inserted in a host germline genome to be endogenized in a host population. Although this time period is unknown, it is basically zero relative to a million years of macroevolution (2.41–6.76 Myr) (Gilbert and Feschotte 2010). Accordingly, we first calculated distances between each eRTBVL-D sequence and the consensus sequences of eRTBVL-A, -B, and -C (fig. 2*A*), which resulted in an average uncorrected distance of 0.260 (fig. 2*B*). Each of these distances was then corrected by subtracting the distance accumulated in host genomes for eRTBVL-D since endogenization, and the latter was approximated as half the distance between orthologous *O. sativa japonica*/*O. meridionalis* eRTBVL-D sequences for each examined locus. The average accumulated distance in host genomes was 0.006. Therefore, the average corrected distance between viral sequences of eRTBVL-D and eRTBVL-A/-B/-C was 0.254 (fig. 2*B*). The corrected distance approximated the accumulated virus–virus distance resulting from the viral substitution rate during the designated time period (fig. 2*A*) (Gilbert and Feschotte 2010). When the time required for a viral sequence inserted in a host germline genome to be endogenized was ignored, the difference between the ages of eRTBVL-D and eRTBVL-A/-B/-C sequences was approximately the same as the divergence time between the viral sequences of the studied eRTBVL-D and eRTBVL-A/-B/-C segments. After dividing by this time (2.25–6.75 Myr), the average lower and upper bounds of long-term substitution rates for the virus were calculated as 3.77 × 10^−8^ and 1.13 × 10^−7^ substitutions/site/year, respectively (fig. 2*B*). Our calculations may have slightly overestimated the actual values because of our assumption about the duration of the endogenization process. Additionally, the virus of eRTBVL-D has been assumed to be the direct ancestor of the viruses of eRTBVL-A, -B, and -C. We cannot exclude the possibility that the virus of eRTBVL-D is an ancient sister of the viruses of eRTBVL-A/-B/-C, although they are very closely related (Chen et al. 2014). Under this possibility, our calculated values would be overestimations. We did not calculate the evolutionary rates during the divergence between the viruses of eRTBVL-A, -B, and -C because of the recombination that occurred among these viruses (Chen et al. 2014).

Using extant viral sequences, PRV short-term substitution rates (2–52 years) were calculated as 1.71 × 10^−4^ to 5.81 × 10^−4^ substitutions/site/year (Yasaka et al. 2014; Guimarães et al. 2015). Thus, long-term substitution rates of PRVs are approximately three to four orders of magnitude slower than short-term rates. Aiewsakun and Katzourakis (2016) recently calculated the viral rate decay speed for the time-dependent rate phenomenon of viruses. In the present study, we simply performed linear regression on the log_10_-transformed substitution rates of PRVs and the corresponding measurement time scales. We obtained a slope (measuring viral rate decay speed) of −0.64 (fig. 2*C*), which is similar to previous estimates of −0.68 (−0.74 to −0.62, 95% highest probability density at viral generic levels) (Aiewsakun and Katzourakis 2016). Thus, our results further support the conclusion that different viral types have a similar viral rate decay speed (Aiewsakun and Katzourakis 2016). Moreover, this consistency suggests that our calculations resulted in a close approximation of the substitution rates.

### Long-Term GRH between Conserved and Divergent PRV Genes

**Fig. 3.**
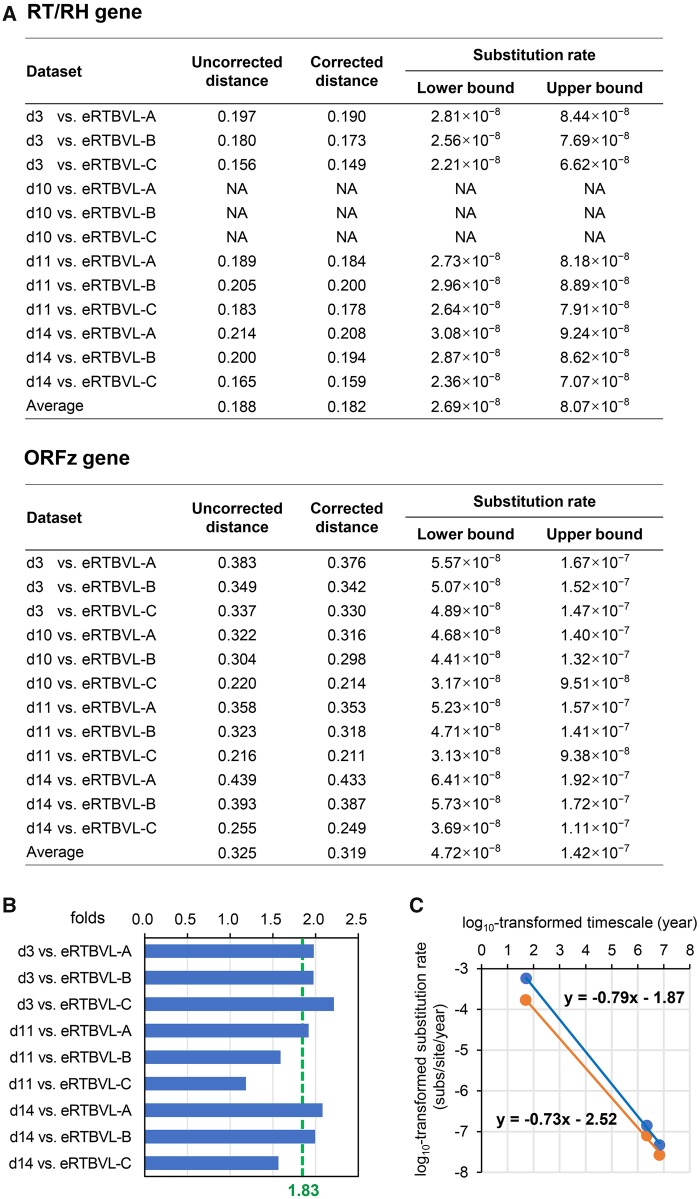
—Long-term GRH between the conserved RT/RH and divergent ORFz genes of PRVs. (*A*) Long-term substitution rates of the RT/RH and ORFz genes of PRVs. Substitution rates were calculated using corrected distances. NA, not available. (*B*) Quantification of the long-term GRH between these two genes. GRH values (fold difference) are displayed on the plots, with green dotted lines indicating the averages. (*C*) Comparison between the rate decay speed of the RT/RH and ORFz genes of PRVs. The plot presents the relationship between gene substitution rates (substitutions/site/year) and the corresponding measurement time scales (years). The log_10_-transformed values underwent a linear regression analysis (orange dots and line for the RT/RH gene, and blue dots and line for the ORFz gene), and the resulting equations are displayed. The short-term data are from a previous study (Yasaka et al. 2014). Note that the short-term substitution rate for the RT/RH gene in the analysis is actually an average value for ORFs I–V of CaMV (the RT/RH gene is located in ORF V), thus the slope for the RT/RH gene is >−0.73.

ORFz, which represents a species-specific divergent gene possibly involved in vector transmission or immune suppression, was covered 4-fold by four eRTBVL-D segments (d3, d10, d11, and d14). Meanwhile, the RT/RH domain in ORFy, which represents the most conserved PRV gene responsible for reverse transcription-mediated genome replication, was covered 3-fold by these segments (fig. 1*A*) (Kunii et al. 2004; Hohn 2013; Hull 2014). Although the identified eRTBVL-D segments only cover about half of the whole viral genomic structures (fig. 1*A*), the coverage of the four segments at the two typical PRV genes allowed us to use them as molecular fossils to reliably examine the long-term GRH between these viral genes. Neutral evolution tests of the respective ORF/domain sequences in each examined eRTBVL-D segment confirmed that these viral gene-derived segments in host genomes have undergone neutral evolution after endogenization, making them suitable molecular fossils (supplementary table S6, Supplementary Material online). We then applied the rate calculation used for whole segment data sets to individual gene segment data sets. We obtained average substitution rates of 4.72 × 10^−8^ to 1.42 × 10^−7^ substitutions/site/year for the viral ORFz gene. Regarding the viral RT/RH gene, the average substitution rates were 2.69 × 10^−8^ to 8.07 × 10^−8^ substitutions/site/year (fig. 3*A*). As expected, these results provide quantitative evidence that long-term substitution rates are heterogeneous across coding regions of viral genomes, thereby supporting the existence of diverse selection pressures on viral genes that differ regarding functional constraints and adaptive plasticity.

Because a given eRTBVL-D segment is associated with a single endogenization time, long-term substitution rates of viral ORFz and RT/RH genes can be directly compared. The alternative relationship between the viruses of eRTBVL-D and eRTBVL-A/-B/-C mentioned earlier likely does not substantially influence this comparison. A comparison of rates calculated for the same eRTBVL-D loci (d3, d11, or d14) revealed a significant long-term GRH between the ORFz and RT/RH genes (*P* value = 1.95 × 10^−3^, Wilcoxon signed-rank test), which was quantified to be average of 1.83-fold (fig. 3*B*). The PR domain in ORFy, which represents another conserved gene involved in processing polyproteins to produce individual functional proteins, was mostly encompassed by the d14 segment (the endogenization time of the d15 segment was unclear) (fig. 1*A*). Therefore, we also determined that the long-term substitution rate of the viral PR gene was 3.99 × 10^−8^ to 1.20 × 10^−7^ substitutions/site/year (supplementary fig. S1*A*, Supplementary Material online). Correspondingly, the long-term substitution rate of the ORFz gene was 1.32-fold that of the PR gene, whereas the long-term substitution rate of the latter gene was 1.44-fold that of the RT/RH gene (supplementary fig. S1*B*, Supplementary Material online). These data suggest that the long-term GRH between differentially conserved PRV genes is unexpectedly low. To the best of our knowledge, this study is the first to estimate the long-term GRH using paleovirological evidence.

### Evolutionary Dynamics of GRH of PRVs

Short-term substitution rates for RTBV have not been reported to date. In a previous study, ORF VI of another PRV, CaMV, was observed to have a short-term substitution rate (average of 5.81 × 10^−4^ substitutions/site/year; 50–52 years) that differed from that of the other ORFs (average of 1.71 × 10^−4^ substitutions/site/year for ORFs I–V), suggesting that ORF VI is the least conserved of the confirmed ORFs of this PRV (Yasaka et al. 2014). This difference means that the short-term substitution rate of ORF VI is 3.40-fold that of ORFs I–V. The gene products of ORF VI have multiple functions, and can act as host range determinants (Schoelz et al. 1986; Haas et al. 2002). The RT/RH gene is located in ORF V of CaMV. Therefore, the short-term GRH between ORF VI and RT/RH genes is >3.40-fold. Additionally, ORFz of the virus of eRTBVL and ORF 4 of RTBV (the counterpart of ORFz in RTBV) are located at the same viral genomic site as ORF VI of CaMV (i.e., immediately after RT/RH domains), and are presumed to have functions similar to those of ORF VI (Hull 1996; Kunii et al. 2004). Nucleotide sequence and encoded amino acid sequence of ORFz of the virus of eRTBVL exhibit no obvious homology with the corresponding sequences of CaMV ORF VI. However, similar amino acid residues in a short key motif (TAV motif) of the ORF VI protein sequence can be recognized in the ORFz protein sequence (Marchler-Bauer et al. 2017). Although short- and long-term data were not obtained for the same viral species, a comparison of their short- and long-term GRHs seems appropriate. Consequently, the long-term GRH estimated in this study may be lower than the short-term GRH, implying a decay in the long-term GRH of PRVs. Although heterogeneity exists between viral gene rates and between viral rates on different time scales, the heterogeneity between the viral GRH on different time scales was untested and unknown. On the basis of our results and previously reported data, we propose that the viral GRH may be heterogeneous on time scales (i.e., time-dependent GRH).

The rate heterogeneity on time scales (millions of years) for a virus can be up to several orders of magnitude, but the GRH heterogeneity on time scales for different viral genes is the same order of magnitude (1.8 vs 3.5). Both the short- and long-term GRHs are low, which may suggest there is a similar dichotomy between core genes and additional genes involved in the adaptation to a particular host for PRVs. We then compared the short- and long-term substitution rates of divergent and conserved PRV genes using our data as well as published data (Yasaka et al. 2014). The ratio of short- to long-term substitution rates for a divergent gene (ORFz and counterparts) was 4.09 × 10^3^ to 1.23 × 10^4^, whereas that for a conserved gene (RT/RH) was < 2.12 × 10^3^ to < 6.36 × 10^3^. Subsequently, we performed linear regression on the log_10_-transformed substitution rates of the two genes and the corresponding measurement time scales. We obtained slopes of −0.79 and > −0.73 for the ORFz and RT/RH genes, respectively (fig. 3*C*). Thus, the lower long-term GRH of PRVs was due to the relatively faster rate decay of divergent genes than of conserved genes during evolution. Consequently, in a viral genome, there is a slight difference in the rate decay speed between the conserved core genes and the divergent additional genes, although all genes exhibit a rate decay during evolution. This slight difference may be useful for further improving the precision of calculations regarding the time-dependent rate phenomenon.

### Possible Drivers and Significance of the Time-Dependence of Viral GRH

There has been some uncertainty regarding whether the time-dependent rate phenomenon is an artefact and is biased because mismodeling can lead to inaccurate time-dependent rate estimates (Soubrier et al. 2012). However, multiple independent observations and strict calibrations support the existence of a time-dependent rate phenomenon in nature (Rocha et al. 2006; Burridge et al. 2008; Henn et al. 2009; Gibbs et al. 2010; Ho et al. 2011; Duchêne et al. 2014; Aiewsakun and Katzourakis 2016). Evidence from paleovirological analyses also support this phenomenon (this study, and Gilbert and Feschotte 2010; Lefeuvre et al. 2011; Suh et al. 2013). Moreover, slightly deleterious nonsynonymous mutations can accumulate rapidly during short-term evolution, but may be swept later, which may represent the biological basis for the time-dependent rate phenomenon (Penny 2005; Rocha et al. 2006). Factors such as purifying selection, transmission bottleneck, highly subdivided population structure, and substitution saturation might act to sweep these slightly deleterious nonsynonymous mutations on the long-term scale (Ho et al. 2007, 2011; Gilbert and Feschotte 2010; Wertheim and Kosakovsky Pond 2011; Aiewsakun and Katzourakis 2016).

The biological basis and drivers described earlier might also contribute to the time-dependent GRH proposed in this study. To explain time-dependent GRH, we need to understand the cause of a slightly faster rate decay for divergent genes than for conserved genes during evolution as concluded above. Regarding short-term scales, divergent genes exhibiting high adaptive plasticity are likely to endure more nonsynonymous mutations than genes under high functional constraints. Additionally, divergent genes endure less nonsynonymous mutations (largely swept) on long-term scales than on short-term scales. These mutations are swept in conserved genes, whereas divergent genes carry more nonsynonymous mutations even after/during sweeping. These processes are actually persistent. Strikingly, a time-dependent rate phenomenon has also been observed in some hosts (Ho et al. 2011). It is possible that the putative time-dependent rate phenomenon of hosts during host–virus coevolution also contributes to the time-dependent GRH of viruses. A decay in the long-term host evolutionary rate may impose a relatively weak selection pressure on weakly conserved viral genes, thereby resulting in the decay of the long-term GRH of viruses. The relatively low long-term GRH of PRVs we observed might be associated with a stable infection and coevolution with rice hosts. Consequently, we hypothesize that the heterogeneity of GRH may be related to the fast adaptation to hosts on short-term scales, and efficient coevolution with hosts on long-term scales.

## Conclusions

In this study, we detected and dated an ancient group of rice EVEs derived from PRVs, which enabled us to explore the long-term GRH of exogenous viruses to study their adaptive evolution. Most segments were 2.41–6.76 Myr old, whereas two segments were traced back to 6.76–15.00 Ma. As expected, an examination of these genomic fossils revealed heterogeneity in long-term substitution rates across different PRV genes and suggested the existence of diverse long-term selection pressures among viral genes. Unexpectedly, the long-term GRH (1.83-fold between the most conserved and divergent genes) was lower than the short-term GRH of PRVs (> 3.40-fold) according to published data. This observation implies that for the determination of the heterogeneity between PRV gene rates the respected time scale matters. The relatively low GRH of PRVs on long-term scales was because of a slightly faster rate decay for divergent genes than for conserved genes during evolution. This difference suggests that although viral sequences exhibit a rate decay during evolution, the speed of this decay differs slightly between the core genes and additional genes of PRVs. It is possible that the adaptive evolution of PRVs may slow down on a long-term scale.

In summary, we examined for the first time the evolutionary dynamics of the GRH of viral genes using genomic fossils. Our results suggest that the GRH of PRVs might be time-dependent, which raises an interesting question concerning GRH dynamics in other viruses, namely, whether the GRH in a viral genome is generally time-dependent. Additional and more detailed studies are required to estimate the GRH of various viral families and groups. In addition to further characterizing viral gene macroevolution, such future research may provide specific insights into the deep coevolution between hosts and viruses. 

## Supplementary Material

Supplementary Data
