# Supplementary material for: Ancient Endogenous Pararetroviruses in Oryza Genomes Provide Insights into the Heterogeneity of Viral Gene Macroevolution
Source: Genome Biol Evol. 2018 Sep 18;10(10):2686–96. doi: 10.1093/gbe/evy207 (PMC6179347; doi:10.1093/gbe/evy207)
Supplement: Supplementary Data [file evy207_supp.zip › Supplementary Fig S1 & Tables S1-S6.pdf]

A

PR gene

Dataset	Uncorrected distance	Corrected distance	Substitution rate	
			Lower bound	Upper bound
d14 vs. eRTBVL-A	0.274	0.268	3.97×10^{-8}	1.19×10^{-7}
d14 vs. eRTBVL-B	0.277	0.271	4.01×10^{-8}	1.20×10^{-7}
d14 vs. eRTBVL-C	0.276	0.270	4.00×10^{-8}	1.20×10^{-7}
Average	0.276	0.270	3.99×10^{-8}	1.20×10^{-7}

RT/RH gene

Dataset	Uncorrected distance	Corrected distance	Substitution rate	
			Lower bound	Upper bound
d14 vs. eRTBVL-A	0.214	0.208	3.08×10^{-8}	9.24×10^{-8}
d14 vs. eRTBVL-B	0.200	0.194	2.87×10^{-8}	8.62×10^{-8}
d14 vs. eRTBVL-C	0.165	0.159	2.36×10^{-8}	7.07×10^{-8}
Average	0.193	0.187	2.77×10^{-8}	8.31×10^{-8}

ORFz gene

Dataset	Uncorrected distance	Corrected distance	Substitution rate	
			Lower bound	Upper bound
d14 vs. eRTBVL-A	0.439	0.433	6.41×10^{-8}	1.92×10^{-7}
d14 vs. eRTBVL-B	0.393	0.387	5.73×10^{-8}	1.72×10^{-7}
d14 vs. eRTBVL-C	0.255	0.249	3.69×10^{-8}	1.11×10^{-7}
Average	0.362	0.356	5.28×10^{-8}	1.58×10^{-7}

B

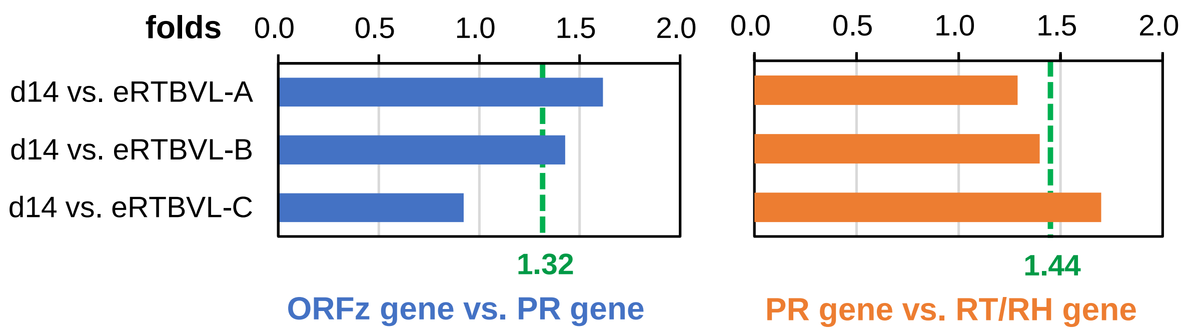

Fig. S1. Long-term GRH between the conserved PR gene and other genes of PRVs. (A) Long-term substitution rates of the conserved PR gene as well as other conserved (RT/RH) and divergent (ORFz) genes revealed using datasets of the d14 segment. Substitution rates were calculated using corrected distances. **(B)** Quantification of the long-term GRH between the PR gene and the two other genes. GRH values (fold difference) are displayed on the plots, with green dotted lines indicating the averages.

Table S1. Information regarding the Oryza genome assemblies examined in this study

Species	Group	Data source	Data version	Note
Oryza sativa japonica	AA-genome	Gramene	IRGSP v1	-
Oryza sativa indica	AA-genome	Molecular Plant	v2Plus	-
Oryza rufipogon	AA-genome	Gramene	OR_W1943	Progenitor of *Oryza sativa*
Oryza glaberrima	AA-genome	Gramene	AGI1.1	-
Oryza barthii	AA-genome	Gramene	v1	Progenitor of *Oryza glaberrima*
Oryza glumaepatula	AA-genome	Gramene	v1.5	-
Oryza meridionalis	AA-genome	Gramene	v1.3	-
Oryza meridionalis	AA-genome	NCBI	LONC00000000.1	Independent source
Oryza punctata	BB-genome	Gramene	v1.2	-
Oryza brachyantha	FF-genome	Gramene	v1.4b	-

Table S2. Detailed results of the orthology analysis, PCR detection, and sequencing of eRTBVL-D loci

ID	*Oryza sativa japonica*		*Oryza sativa indica*		*Oryza rufipogon*		*Oryza glaberrima*		*Oryza barthii*		*Oryza glumaepatula*		*Oryza meridionalis*		*Oryza punctata*		*Oryza brachyantha*	
	**Orthology analysis**	**PCR & Seq**	**Orthology analysis**	**PCR & Seq**	**Orthology analysis**	**PCR & Seq**	**Orthology analysis**	**PCR & Seq**	**Orthology analysis**	**PCR & Seq**	**Orthology analysis**	**PCR & Seq**	**Orthology analysis**	**PCR & Seq**	**Orthology analysis**	**PCR & Seq**	**Orthology analysis**	**PCR & Seq**
d1	presence	presence	presence	presence	presence	presence	presence	presence	presence	presence	presence	presence	presence	presence	absence	absence	absence	absence
d2	presence	no data	presence	no data	presence	no data	delete	no data	delete	no data	delete	no data	presence	no data	absence	no data	absence	no data
d3	presence	presence	presence	presence	presence	presence	presence	presence	presence	presence	presence	presence	presence	presence	absence	absence	absence	absence
d4	presence	presence	presence	presence	presence	presence	unknown	presence	presence	presence	presence	presence	unknown	presence	absence	absence	absence	absence
d5	presence	presence	presence	presence	presence	presence	unknown	presence	presence	presence	presence	presence	unknown	presence	absence	absence	absence	absence
d6	presence	no data	presence	no data	presence	no data	presence	no data	presence	no data	presence	no data	presence	no data	absence	no data	absence	no data
d7	presence	no data	presence	no data	presence	no data	presence	no data	presence	no data	presence	no data	presence	no data	absence	no data	absence	no data
d8	presence	presence	presence	presence	presence	presence	presence	presence	presence	presence	unknown	presence	presence	presence	absence	absence	absence	absence
d9	presence	presence	presence	presence	presence	presence	unknown	presence	presence	presence	presence	presence	unknown	unknown	absence	absence	absence	absence
d10	presence	presence	presence	presence	presence	presence	presence	presence	presence	presence	presence	presence	presence	presence	absence	absence	absence	absence
d11	presence	presence	presence	presence	presence	presence	presence	presence	presence	presence	presence	presence	presence	presence	absence	absence	absence	absence
d12	presence	no data	presence	no data	presence	no data	presence	no data	presence	no data	presence	no data	presence	no data	absence	no data	absence	no data
d13	presence	presence	presence	presence	presence	presence	unknown	presence	presence	presence	presence	presence	presence	presence	absence	absence	absence	absence
d14	presence	presence	presence	presence	presence	presence	presence	presence	presence	presence	presence	presence	presence	presence	absence	absence	absence	absence
d15	presence	presence	presence	presence	presence	presence	presence	presence	presence	presence	presence	presence	unknown	unknown	absence	absence	absence	absence

Parts of the results of d1-d3, d10, d11, d14, and d15 in *O. sativa* and *O. rufipogon* were extracted from Chen et al. (2014). No data: PCR was unperformed or failed due to no suitable primer pairs (low primer specificity). Unknown: orthology analysis was hindered by the uncoverage of the target region in genome assemblies (assembly gap/uncharacterized bases 'N' in genome data).

Table S3. Information regarding Oryza species accessions for PCR and sequencing confirmatio

Accession	Species	Group	Original place	Note
Nipponbare	*Oryza sativa japonica*	AA-genome	Japan	-
93-11	*Oryza sativa indica*	AA-genome	China	-
W0107	*Oryza rufipogon*	AA-genome	India	Progenitor
IRGC103777	*Oryza glaberrima*	AA-genome	Mali	-
W1588	*Oryza barthii*	AA-genome	Cameroun	Progenitor
W1169	*Oryza glumaepatula*	AA-genome	Cuba	-
W1625	*Oryza meridionalis*	AA-genome	Australia	-
W1514	*Oryza punctata*	BB-genome	Kenya	-
W1402	*Oryza brachyantha*	FF-genome	Sierra Leone	-

Table S4. Details regarding the primers used for PCR and sequencing

Target ID	Primer name	Sequence	Note
d1 and d3	JaE1-4-1	CGTGTATTTAAAGAGACACATTCACAATTA	Forward
	JaE1-4-3	GTGCAAGAGGCTAGAAACAAGA	Forward
	JaE1-4-4	TATCATACTGTGGCATCATGGGTTC	Reverse
d4 and d5	Ch2-3.1F	CAGACTCATCCAAAACTGCT	Forward
	Ch2-3.1R	TATCAATCATTCAGGGGTGG	Reverse
	Ch2-3.1F2	CACTATAGCTGTACTGTAATGAC	Forward
	Ch2-3.1R2	GCAAGCCTAATAGTCTACAAG	Reverse
	Ch2-3.1SF2	GTATGCAGACTCATCCAAAAC	Forward
d8	Ch4-3.1F	AGAAAGCCATTCAGCCCTCG	Forward
	Ch4-3.1R	CATCGCCACCGGAAACTATC	Reverse
	Ch4-3.1SF2a	GACTGTGTAGTAGTTAGAG	Forward
	Ch4-3.1SF2b	GCTCTCTTCCTCACTCCTAG	Forward
	Ch4-3.1SR2a	TGATTGTGCGACACCGACG	Reverse
	Ch4-3.1SR2b	GAGCTTCTCTCCTCTCATC	Reverse
	Ch4-3.1SF3b	GATCTGCCATTGTGCGAGC	Forward
	Ch4-3.1SR3b	CTGGTGTGTTCTGTACAAGTG	Reverse
	Ch4-3.1F2	ACAGAAGGGATAGCACGAGG	Forward
	Ch4-3.1R2	GCCGTTTGTCCAATTGAGAT	Reverse
	Ch4-3.1F3	ACCCCCGAGCTTACAGACTT	Forward
d9	Ch5-0.1F	GATTGTATTCATGCTGCAGC	Forward
	Ch5-0.1R	AGTACAGCAGGATTAGCAAG	Reverse
d10	Ch7-5-1	AGGAAGTGCCACTACATGGT	Forward
	Ch7-5-2	CTGAACAGATAAACTTGCATACAAGC	Reverse
	Ch7-5-3	CATACAAGCTTTCTTCTTACCTTAC	Forward
	Ch7-5-4	ATTGATGCATATATAACATCGGCTCG	Reverse
d11	JaE7-5F	GAGTGGTGTCTTTGTTGTTTCTC	Forward
	JaE7-5-1&SF2	CATCAAATTCCTCATYATAAGCC	Forward
	JaE7-5SR2	GGTATTGTACTGTGGCCTCC	Reverse
	JaE7-5-4	CACCACAGTGAAGCAATATGTGGAT	Reverse
	JaE7-5R	TAGTCCCTTAGGTGCCTAGC	Reverse
	JaE7-5SF2b	CAATTGAGGGGAGGCTTCTTC	Forward
	JaE7-5SF2c	CACCATCTAAGGGATCCATC	Forward
	Ch7-6-1	GTAAAAGGGACCGTGGACTT	Forward
	Ch7-6-2	GGTCAGGACAGATTGTGAATGTA	Reverse
	Ch7-6-3	TAACTCTAAAATGAGCGGATTGAAAC	Forward
	Ch7-6-4	CTCTATCTCCCAACCCAAGA	Reverse
	JaE7-6F2	AAGTCACGGACATCTGGAGG	Forward
	JaE7-6R2	GGGCCTGTCTTAACGTGGTA	Reverse
	JaE7-6-1	TCACAATCTGTCCTGACCGT	Forward
	JaE7-6-4	TCAATCCGCTGCATTTTAGA	Reverse
d13	Ch10-0.1-1	GCATGWTTGGCCACTATGTG	Forward
	Ch10-0.1-2	GAAAATATGTACAAGCATACATAC	Reverse
	Ch10-0.1-4	CTATCAATGGCTTAACTTCAATG	Reverse
d14	JaE11-2-1	TATCACACGCTGTCTCCCTA	Forward
	JaE11-2-2	GGAATTTGAAATGTAATATCCGT	Reverse

	JaE11-2-3	CAGCAGAAGCCTACTCCC	Forward
	JaE11-2-SF	CGAGGCGTCCAGTTCTAATC	Forward
	JaE11-2-4	GGATATTTACTTTAGTGTCCGGT	Reverse
	JaE11-2-3R	CCAAACTCTATGGCGATGGT	Reverse
	JaE11-2-1F	CTCGCCTATCTCTAGCAGGC	Forward
	JaE11-2-4R	GCTGCCCAGTACTGAAGGAG	Reverse
	JaE11-2-1Fa	CACAATTGGTCCAAGGTTCC	Forward
	JaE11-2-4Ra	AGCAGCGGATATTGTGCATA	Reverse
d15	Ch11-4-1	GGTTGGCCCTTCTTCCTTTAT	Forward
	Ch11-4-1R	GGCATGCCCACACATACATA	Reverse
	JaE11-5SF2	CTTCATACCTGCAAAACAGG	Forward
	JaE11-5SF3	AATTGAAATATCTCTGACCAT	Forward
	JaE11-5SF4	GCCAAAATCCCGCTTATCAGTC	Forward
	JaE11-5SR2	GCATACATCAATGAGACTAG	Reverse
	Ch11-4-2R	ATGTCCGATAGAGACGACCC	Reverse
	Ch11-4-4	GCGAAGGCTACGCTACTAAC	Reverse
	Ch11-4-2	GCCGCTGGCTCTTCATAATA	Reverse
	Ch11-4-3	CCAGGATACCACATTGCACA	Forward
	Ch11-4-1F	TTCTTCATACCTGCAAAACAGG	Forward
	Ch11-4-2	ATGCCAGTTGCAGAAAATATTGAGAC	Reverse
	Ch11-4-3	CTCATCTATTAATATCTAGTCTCATTGATG	Forward
	JaE11-5R	GCTAAGACGACGAGGGCAG	Reverse

Table S5. Comparison of distance calculations using raw and consensus sequences of eRTBVL-A, -B, and -C

Dataset	Raw sequence	Consensus sequence	Difference
d3 vs. eRTBVL-A	0.289 ± 0.003	0.279	0.010
d3 vs. eRTBVL-B	0.270 ± 0.002	0.256	0.014
d3 vs. eRTBVL-C	0.241 ± 0.003	0.237	0.004
d10 vs. eRTBVL-A	0.343 ± 0.005	0.318	0.025
d10 vs. eRTBVL-B	0.321 ± 0.006	0.304	0.017
d10 vs. eRTBVL-C	0.224 ± 0.003	0.222	0.002
d11 vs. eRTBVL-A	0.271 ± 0.004	0.267	0.004
d11 vs. eRTBVL-B	0.270 ± 0.005	0.261	0.009
d11 vs. eRTBVL-C	0.207 ± 0.003	0.200	0.007
d14 vs. eRTBVL-A	0.297 ± 0.003	0.288	0.009
d14 vs. eRTBVL-B	0.288 ± 0.003	0.272	0.016
d14 vs. eRTBVL-C	0.229 ± 0.002	0.221	0.008
Average	0.271 ± 0.004	0.260	0.010

Here, all the numerical values were rounded to 3 decimal places to show the standard deviation and difference

Table S6. Results of tests of neutral evolution conducted for the four long eRTBVL-D segmer

**Dataset**	**Test**	**Significance**	**Note**
d3 (RT/RH domian)	*dN*/*dS* ratio test (sites)	all $p > 0.48$	No significant selection
	dN/*dS* ratio test (pairs)	all $p > 0.11$	No significant selection
	Tajima's D test	$p > 0.10$	No significant selection
	Fu and Li's tests (D*)	$p > 0.10$	No significant selection
	Fu and Li's tests (F*)	$p > 0.10$	No significant selection
d3 (ORFz)	*dN*/*dS* ratio test (sites)	all $p > 0.51$	No significant selection
	dN/*dS* ratio test (pairs)	all $p > 0.33$	No significant selection
	Tajima's D test	$p > 0.10$	No significant selection
	Fu and Li's tests (D*)	$p > 0.10$	No significant selection
	Fu and Li's tests (F*)	$p > 0.10$	No significant selection
d10 (ORFz)	*dN*/*dS* ratio test (sites)	all $p > 0.41$	No significant selection
	dN/*dS* ratio test (pairs)	all $p > 0.50$	No significant selection
	Tajima's D test	$p > 0.10$	No significant selection
	Fu and Li's tests (D*)	$p > 0.10$	No significant selection
	Fu and Li's tests (F*)	$p > 0.10$	No significant selection
d11 (RT/RH domian)	*dN*/*dS* ratio test (sites)	all $p > 0.66$	No significant selection
	dN/*dS* ratio test (pairs)	all $p > 0.19$	No significant selection
	Tajima's D test	$p > 0.10$	No significant selection
	Fu and Li's tests (D*)	$p > 0.10$	No significant selection
	Fu and Li's tests (F*)	$p > 0.10$	No significant selection
d11 (ORFz)	*dN*/*dS* ratio test (sites)	all $p > 0.63$	No significant selection
	dN/*dS* ratio test (pairs)	all $p > 0.17$	No significant selection
	Tajima's D test	$p > 0.10$	No significant selection
	Fu and Li's tests (D*)	$p > 0.10$	No significant selection
	Fu and Li's tests (F*)	$p > 0.10$	No significant selection
d3 (whole length)	Tajima's D test	$p > 0.10$	No significant selection
	Fu and Li's tests (D*)	$p > 0.10$	No significant selection
	Fu and Li's tests (F*)	$p > 0.10$	No significant selection
d11 (whole length)	Tajima's D test	$p > 0.10$	No significant selection
	Fu and Li's tests (D*)	$p > 0.10$	No significant selection
	Fu and Li's tests (F*)	$p > 0.10$	No significant selection
d10 (whole length)	Tajima's D test	$p > 0.10$	No significant selection
	Fu and Li's tests (D*)	$p > 0.10$	No significant selection
	Fu and Li's tests (F*)	$p > 0.10$	No significant selection
d14 (whole length)	Tajima's D test	$p > 0.10$	No significant selection
	Fu and Li's tests (D*)	$p > 0.10$	No significant selection
	Fu and Li's tests (F*)	$p > 0.10$	No significant selection

Tests on the individual gene datasets of d14 were not performed, because large deletions in the orthologous sequences result in inadequate data for tests.
