# Supplementary material for: Ancient Endogenous Pararetroviruses in Oryza Genomes Provide Insights into the Heterogeneity of Viral Gene Macroevolution
Source: Genome Biol Evol. 2018 Sep 18;10(10):2686–96. doi: 10.1093/gbe/evy207 (PMC6179347; doi:10.1093/gbe/evy207)
Supplement: Supplementary Data [file evy207_supp.zip › SUPPLEMENTARY MATERIAL.docx]

# Supplementary material

**Fig. S1. Long-term GRH between the conserved PR gene and other genes of PRVs. (A)** Long-term substitution rates of the conserved PR gene as well as other conserved (RT/RH) and divergent (ORFz) genes revealed using datasets of the d14 segment. Substitution rates were calculated using corrected distances. **(B)** Quantification of the long-term GRH between the PR gene and the two other genes. GRH values (fold difference) are displayed on the plots, with green dotted lines indicating the averages.

**Table S1. Information regarding the *Oryza* genome assemblies examined in this study**

**Table S2. Detailed results of the orthology analysis, PCR detection, and sequencing of eRTBVL-D loci**

**Table S3. Information regarding *Oryza* species accessions for PCR and sequencing confirmation**

**Table S4. Details regarding the primers used for PCR and sequencing**

**Table S5. Comparison of distance calculations using raw and consensus sequences of eRTBVL-A, -B, and -C**

**Table S6. Results of tests of neutral evolution conducted for the four long eRTBVL-D segments**

**Dataset S1. Sequence alignments for the four long eRTBVL-D segments**
